# New Pharmacological Therapies in the Treatment of Epilepsy in the Pediatric Population

**DOI:** 10.3390/jcm13123567

**Published:** 2024-06-18

**Authors:** Karolina Daniłowska, Natalia Picheta, Dominika Żyła, Julia Piekarz, Katarzyna Zych, Paulina Gil-Kulik

**Affiliations:** 1 Student’s Scientific Society of Clinical Genetics, Medical University of Lublin, 20-080 Lublin, Poland; karolina02812@gmail.com (K.D.); natalia.picheta2812@gmail.com (N.P.); dominika.zyla2211@gmail.com (D.Ż.); piekarzjulia1@gmail.com (J.P.); katarzyna.zych.98@gmail.com (K.Z.); 2 Department of Clinical Genetics, Medical University of Lublin, 20-080 Lublin, Poland

**Keywords:** epilepsy in children, pediatric epilepsy, pharmacological treatment, antiseizure drugs, seizure control

## Abstract

Epilepsy is a disorder characterized by abnormal brain neuron activity, predisposing individuals to seizures. The International League Against Epilepsy (ILAE) categorizes epilepsy into the following groups: focal, generalized, generalized and focal, and unknown. Infants are the most vulnerable pediatric group to the condition, with the cause of epilepsy development being attributed to congenital brain developmental defects, white matter damage, intraventricular hemorrhage, perinatal hypoxic-ischemic injury, perinatal stroke, or genetic factors such as mutations in the Sodium Channel Protein Type 1 Subunit Alpha (*SCN1A*) gene. Due to the risks associated with this condition, we have investigated how the latest pharmacological treatments for epilepsy in children impact the reduction or complete elimination of seizures. We reviewed literature from 2018 to 2024, focusing on the age group from 1 month to 18 years old, with some studies including this age group as well as older individuals. The significance of this review is to present and compile research findings on the latest antiseizure drugs (ASDs), their effectiveness, dosing, and adverse effects in the pediatric population, which can contribute to selecting the best drug for a particular patient. The medications described in this review have shown significant efficacy and safety in the studied patient group, outweighing the observed adverse effects. The main aim of this review is to provide a comprehensive summary of the current state of knowledge regarding the newest pharmacotherapy for childhood epilepsy.

## 1. Introduction

Epilepsy is defined as a brain disorder characterized by a persistent predisposition to generate epileptic seizures in combination with the neurobiological, cognitive, psychological, and social implications of the condition. An epileptic seizure is a temporary occurrence of symptoms caused by abnormal neuronal activity in the brain [1,2]. A detailed division of epileptic seizures has been proposed by the International League Against Epilepsy (ILAE), which reflects the relevance of seizure type, epilepsy type, syndromes, and etiology, helping to apply the division in clinical practice. ILAE divides seizures into three main groups: focal, generalized, and unknown, and then into subgroups on the basis of motor and nonmotor onset. Epilepsies were classified by ILAE into focal, generalized, combined generalized and focal, and unknown epilepsies [2,3,4].

Epidemiologically, epilepsy has an unequal population distribution, with ∼80% of those affected living in low- and middle-income countries. (high-income countries—48.86 per 100,000 person years, low- and middle-income countries—138.99 per 100,000 person years) [4,5]. The highest risk of epilepsy occurs in infants due to genetic, metabolic, and obstetric causes [5]. Children are more likely to experience seizure-related accidents than adults. The most common are minor accidents like fractures, burns, and drownings, with falls being the most dangerous [4].

Based on data received from European studies, the etiology of epilepsy may be defined in approximately 54% of the general pediatric population and 64% of children who had symptoms in the first year of life. Structural etiology is common in patients who have it identified before the seizure presentation. Among them, the most common are congenital brain malformations, white matter injuries, intraventricular hemorrhage, hypoxic ischemic perinatal injuries, or perinatal stroke [6,7]. The genetic basis of epilepsy may affect as many as two-thirds of epileptic tendencies in the general pediatric population. Among the genetic causes listed are monogenic mutations, polygenic mutations, and multifactorial pathologies [8]. The most significant known monogenic factor is a mutation in the *SCN1A* gene and its brain voltage-gated sodium channel NaV1.1 loss-of-function variants, causing Dravet syndrome—a severe drug-resistant epilepsy and intellectual disability. Different types of *SCN1A* mutations may cause other types of midler phenotypes of genetic epilepsy [9]. Significant gene mutations that may be associated with epilepsy also include Proline-rich transmembrane protein 2 (*PRRT2*), Sodium Channel, Voltage-gated, Type III, Alpha Subunit *SCN3A*, Potassium Voltage-Gated Channel Subfamily C Member 1 and 2 (*KCNC1* and *KCNC2*), Voltage-dependent Calcium Channel Subunit Alpha-2/delta-1 (*CACNA2D1*), Synaptosome Associated Protein 25 (*SNAP25*), Spectrin Beta, Non-Erythrocytic 1 (*SPTBN1*), Cyclin Dependent Kinase Like 5 (*CDKL5*), and many others. A common feature of most genetic epilepsies is their drug resistance. This is also the main reason for the need for treatment to be more intensive than in the adult population, requiring frequent use of different therapeutic options before finding the one suitable for a particular patient [10,11]. Neonatal seizures are a type of epileptic seizure defined as occurring with no potentially causative clinical conditions or outside the estimated interval for the occurrence of acute symptomatic seizures. Their presence should induce further diagnosis of potential causes of structural and vascular malformations or ischemic lesions in the brain, as well as genetic disorders [12]. Status epilepticus (SE) is a term introduced by ILAE in 2015. It highlights the fact that prolonged (lasting about 5 min) seizures can continue and cause severe consequences. It suggests that seizures lasting over 5 min should be treated medically in order to terminate them and stop them from lasting longer than 30 min, which could lead to long-term complications. It is important to consider the etiology of SE in order to apply the most suitable treatment. Considering and excluding potentially treatable causes like infections or metabolic causes should be the first step [13]. The worst form of SE is new-onset refractory status epilepticus (NORSE); the term refers to cases of super-refractory status epilepticus (SRSE) without a previous history of epilepsy and with no known cause. Seizures are at first brief and infrequent, and their frequency increases over several hours to days (up to hundreds per day) and develops to SE, which usually requires ICU admission and anesthesia [14]. Epilepsy syndromes such as Dravet syndrome, Lennox-Gastaut syndrome, and sunflower syndrome are characteristic of the pediatric population. Dravet syndrome (DS) is a type of encephalopathy characterized by multiple types of seizures, often triggered by different factors such as elevated temperature, infections, or light. The seizures are resistant to standard pharmacological treatment. Normal cognitive development at first, which is followed by stagnant development at the age of 1 to 4 years old, is common. Other characteristic features of DS are intellectual disability (ID) in most of the patients, a higher incidence of autism spectrum disorders (ASD), and progressive walking impairment occurring in early school. The most common cause of death in patients with DS is sudden, unexpected death in epilepsy (SUDEP) and status epilepticus (SE) [15]. Lennox-Gastaut syndrome (LGS) is defined by a triad of multiple drug-resistant seizure types, a specific interictal electroencephalographic (EEG) pattern exhibiting bursts of either slow spike-wave complexes (SSW) or generalized paroxysmal fast activity (GPFA), and intellectual disability (ID). The interictal EEG pattern of SSW, which is associated with LGS, is not pathognomonic for the disorder [16,17]. Sunflower syndrome is a rare photosensitive epilepsy typically marked by stereotypic seizures and light attraction. A typical description of a seizure is an individual looking toward a light and waving their hand in front of their eyes. Those episodes are associated with generalized spike-and-wave discharges in the EEG [18].

Different antiseizure drugs (ASDs) are used, depending on the country or even individual hospitals, to treat epilepsy in children. European protocols and guidelines are based on Advanced Life Support (ALS) and Advanced Pediatric Life Support (APLS) documents, while in the United States, they are based on American Epilepsy Society guidelines. First-line ASMs are benzodiazepines; the most commonly used are lorazepam, midazolam, and diazepam. Second line therapies include phenobarbital, phenytoin, levetiracetam, and sodium valproate. There is disagreement about the specific drug, dose, and route of administration [13]. In severe drug-resistant epilepsies, surgical methods can be used, although there is no evidence to support the idea that early surgical intervention in appropriately selected patients with DRE offers the best possible opportunity to avoid lifelong disability and premature death. The high risk associated with performing such surgeries gives reason to continually seek new treatment options using less invasive pharmacological treatments [19].

Because the topic is still not sufficiently described in the literature, we conducted a study describing and comparing different pharmacological treatment options for epilepsy and SE in the pediatric population. Based on the collected literature, we decided to summarize existing and newer treatments, their effectiveness, and their possible use in specific seizure types and syndromes. 

## 2. Materials and Methods

We performed a narrative review of the medical literature about the pharmacological treatment of epilepsy in the pediatric population. We searched Pubmed, Google Scholar, and Embase databases for studies from January 2018 to January 2024; three studies were older than this time period (2005, 2010, 2013). Such a time frame was chosen by the authors to make the information on drug therapy options as up-to-date as possible but sufficiently comprehensive on the topic discussed. The keywords used for the search included “epilepsy in children”, “pediatric epilepsy”, “pharmacological treatment”, “antiseizure drugs”, and “seizure control” in order to find as wide a range of articles covering the topic discussed as possible. As inclusion criteria, we accepted articles in English on therapies proven in case–control studies and on patients diagnosed with epilepsy. The number of articles finally taken into account was 104. We excluded case studies, conference abstracts only, duplicate papers, and preprint articles.

## 3. Pharmacological Agents

### 3.1. Cenobamate

Cenobamate (CNB) is a monocarbamate that blocks sodium receptors and modulates the GABA-A receptor [20]. It has been approved by the US Food and Drug Administration (FDA) and the European Medicines Agency (EMA) as a new ASD. However, only for the treatment of focal-onset seizures in adults [20,21,22]. CNB is still not approved for use in children and adolescents due to limited experience with this drug [20,23]. Taking into account the promising prospects for the use of CNB in the treatment of epilepsy in children, research is being conducted to increase knowledge about the possibilities of using this substance in the pediatric population.

The CNB mechanism of action is related to sodium and GABA channels [24]. It inhibits voltage-gated sodium currents. There is an enhancement of fast and slow inactivation of sodium channels, as well as inhibition of the non-activating permanent component of the sodium channel current. The advantage of CNB is that it regulates the excitability of principal neurons without damaging inhibitory interneurons. It is possible due to two types of impacts. A small effect on the peak component of transient sodium currents induced by short depolarizing step pulses and a strong inhibition of the non-inactivating persistent component of sodium currents [25,26]. Moreover, CNB is an allosteric modulator of the GABA ion channel. It similarly affects all GABA-A receptors with α1β2γ2 or α2-6β3γ2 subunits [24].

One study describes the evaluation of CNB in the treatment of drug-resistant epilepsy (DRE) in children. Sixteen pediatric patients aged 12.08 to 17.67 years (median 15.05) with disease durations ranging from 6.33 to 16.42 years (median 11.73) were investigated. The causes of epilepsy were considered to be abnormalities in cortical development, residual brain damage after a heart attack, asphyxia, encephalitis, and genetic and autoimmune diseases. In some cases, the cause was unknown. Oral CNB was administered once a day. The initial dose ranged from 6.25–25 mg (median 12.5) and 0.12–0.36 mg/kg/d (median 0.22). In most patients, the dose was increased every 2 weeks to a maximum dose of 0.89–7 mg/kg/d (median 3.1), resulting in a total daily dose of 50–400 mg (median 182.81). The side effects were not serious enough to require the discontinuation of CNB. The treatment effect was assessed after 56–314 days (median 168.5). Seizure freedom was observed in five patients (31.3%). A reduction of seizures by more than 50% was noticed in six patients (37.5%), while in another four patients, a reduction of seizures by less than 50% was observed. In one case, there was an increase in the frequency of seizures. The study shows that CNB has a positive effect on reducing DRE attacks in pediatric patients [23].

Another study presents the use of CNB in pediatric patients with focal-onset epilepsy. It included 21 patients aged 10 to 18 years (median 15.9). The doses of CNB were 209.8 mg and 200 mg. Patients weighing less than 50 kg were included, and the doses were 4 mg/kg/d and 4.32 mg/kg/d. Patients were followed up for 46–496 days (mean 254). The frequency of seizures was reduced by 93.7%. A reduction of episodes by at least 50% was observed in 13 patients and by at least 75% in 11 of them (52.4%). Moreover, four patients (19%) achieved seizure freedom. Unfortunately, in two patients (9.5%), the frequency of attacks increased. According to the interpretation of the above-described study, CNB reduces the incidence of refractory focal-onset epilepsy in children [20]. Furthermore, in a study on the use of CNB in the treatment of focal-onset epilepsy, the pediatric group consisted of 13 patients aged 12 to 17 years. The daily dose of CNB used was 50–300 mg/d (median 204). The follow-up period was 2–15 months (median 9). A total of 61.5% of pediatric patients noted an improvement in the frequency of seizures and thus, a reduction in their number by at least 50%. No serious side effects have been reported. According to the obtained results, the effectiveness and safety of CNB use in the pediatric group were confirmed [21].

According to the above-mentioned studies, CNB significantly reduces the incidence of epileptic seizures in children. Moreover, emerging side effects do not constitute an indication to discontinue the medication. In individual cases, only the dose of CNB had to be reduced. The side effects that occur are not considered serious effects of the therapy. The most common were mainly somnolence and fatigue [20,23]. Table 1 presents the results of the effectiveness of CNB in reducing seizures.

### 3.2. Fenfluramine

Fenfluramine (FFA) is an amphetamine derivative that promotes the release of serotonin (5-HT) and also blocks its neuronal reuptake. The FDA has approved the use of FFA for the treatment of seizures associated with Dravet syndrome (DS) in patients 2 years of age and older, and the EMA has recognized FFA as a safe add-on therapy to other ASDs in patients 2 years of age and older. Moreover, the FDA and EMA have also approved FFA for the treatment of seizures associated with Lennox-Gastaut syndrome (LGS) from the age of 2 years [28].

The anticonvulsant mechanism of FFA is associated with its impact on the serotonergic system. FFA increases the level of extracellular 5-HT, resulting in the activation of 5-HT receptors. By interacting with the serotonin transporter, it inhibits 5-HT uptake. Moreover, FFA causes the release of 5-HT outside the cell. It occurs using 5-HT carriers, ultimately leading to an increase in the extracellular level of 5-HT [29]. The mechanism described above is an indirect effect of FFA. There is also a direct route of activation of 5-HT receptors, which is responsible for FFA metabolites such as D- and L-norfenfluramine (norFFA) [30]. Direct and indirect effects lead to an increase in 5-HT as well as the frequency and amplitude of spontaneous inhibitory postsynaptic currents. Therefore, GABAergic neurotransmission in inhibitory interneurons is enhanced [29].

One of the studies assessing the treatment of DS using FFA as an adjunct to Stripentol therapy (STP) involved 87 pediatric patients, mainly aged 6–19. A total of 44 of them received a placebo, and 43 received FFA. The starting dose of FFA was 0.2 mg/kg/d and was gradually increased over 3 weeks to a final dose of 0.4 mg/kg/d. Patients received the drug orally twice daily in the form of fenfluramine hydrochloride containing 2.2 mg/mL FFA. FFA use was maintained for over 12 weeks. Patients experienced clinically significant (50% or more) reductions in monthly convulsive seizure frequency (MCSF), as well as significantly longer seizure-free periods (3–105, median 22). Additionally, the most common treatment-related side effects included decreased appetite, fever, fatigue, and diarrhea [31]. Based on the results of another study on the use of FFA in pediatric patients with DS, similar conclusions as above can be reached regarding the effect of this drug on the frequency of seizures. In this sample, patients suffered from tonic, clonic, tonic-atonic, generalized tonic-clonic, hemiclonic, or focal seizures with motor symptoms. A total of 119 patients aged 2 to 18 years were assigned to subsequent experimental groups. A total of 40 of them received a placebo, 39 patients received FFA 0.2 mg/kg/d, and 40 patients received 0.7 mg/kg/d. The frequency of seizures after 14 weeks of treatment is statistically significantly reduced. In the group with FFA 0.7 mg/kg/d, it was on average 74.9%, and in the group with FFA 0.2 mg/kg/d, it was 42.3%. Compared to placebo, patients with FFA 0.7 mg/kg/d had a 52.3% greater reduction in MCSF, and patients with FFA 0.2 mg/kg/d had a 32.4% greater reduction. Interestingly, in the group with a higher dose of FFA, the median of the longest seizure-free period was 25 days, while in the group with a lower dose of FFA, it was 15 days [32]. The side effects in the presented trial are consistent with those occurring in the previously described study. In both cases, patients did not develop any side effects related to the cardiovascular system. Additionally, the use of FFA reduced the frequency of using ASDs [31,32]. Similar conclusions were made in one Italian study on DS, which involved 52 pediatric patients with an average age of 8.6 years. Each of them was a carrier of the *SCN1A* genetic variant. FFA was used as an additional drug at an average dose of 0.46 mg/kg/d, and the median follow-up time was 9 months. In the described study, 32 patients experienced a minimum 50% reduction in seizure frequency [33]. Referring to the influence of FFA on body weight and height, a minimal influence of FFA on the rate of growth and weight gain was also proven. The study covers 279 patients treated with FFA for 12 months or longer and 128 patients treated with FFA for 24 months or longer. The average age was 10 years, and the average BMI was 18 mg/m^2^ [34].

Moreover, one meta-analysis compared the effectiveness, tolerability, and global functioning of FFA with other ASM therapies. FFA had a higher seizure response rate at 0.8 mg/kg/d than at 0.2 mg/kg/and, compared to other ASMs. However, FFA, compared to cannabidiol, had a higher risk of side effects among pediatric participants. Despite this, FFA was associated with higher rates of improvement [35].

In addition to the use of FFA in the treatment of DS, there is also a study on the effectiveness of this drug in LGS therapy. According to it, FFA can be a safe and effective long-term therapy. In an open-label extension (OLE) of a phase 3 randomized clinical trial (RCT), there was a mixed group of adult and pediatric patients aged 2–35 years. A total of 168 of 247 patients were between 2 and 18 years of age. The inclusion requirement was the current fulfillment of the primary study criteria. These included tonic or atonic seizures, as well as the onset of seizures before the age of 11. During the initial 14-day transition period from RCT to OLE, all patients were titrated with FFA to 2 mg/kg/d. According to the study plan, they remained at a dose of 2 mg/kg/d for 1 month. Then, taking into account tolerance, the FFA dose was increased to 7 mg/kg/d. Efficacy was assessed at 1, 2, 3, 6, 9, and 12 months, with combined follow-up visits at 3 and 6 months after the last dose. Patients were treated for an average of 364 days. In the pediatric group, a reduction in the frequency of drop and non-drop seizures was observed by an average of 25.6% and 48.9%, as well as a reduction in the frequency of generalized tonic-clonic seizures by an average of 48.8%. The most common side effects during therapy included decreased appetite and fatigue. Any cardiovascular events among patients were excluded [36].

Based on information from one study, FFA is effective as an adjunct therapy for epilepsy associated with sunflower syndrome. Patients with a mean age of 10 years and 4 months (cohort 1) and 13 years and 4 months (cohort 2) were stratified and included in the study. FFA was taken twice a day at a dose of 0.2 mg/kg/d for the first 14 days. The dose was then increased by 0.2 mg/kg/d every 2 weeks to reach a maximum dose of 0.7 mg/kg/d. The maximum dose was maintained for over 2 years. Among cohort 1, an average 79% reduction in the frequency of handwaving episodes (HWE) was observed compared to baseline, and in cohort 2, an average reduction of 33% was observed. The most common side effects, as in the case of GS and LGS, included decreased appetite and fatigue [37]. Table 2 lists studies proving the effectiveness of various doses of fenfluramine in reducing epileptic seizures.

To summarize the studies cited above, FFA reduces the frequency of seizures in pediatric patients with DS on its own and as an additional substance. Moreover, it is also possible to use this medicine in LGS or sunflower syndrome. Side effects are not serious and most often include decreased appetite or fatigue.

### 3.3. Diazepam and Midazolam Intranasally

Diazepam is a drug commonly used for epileptic seizures, including in pediatric patients. It belongs to a group of benzodiazepines that bind at an allosteric site at the boundary of the alpha and gamma subunits on ion channels on the GABA-A receptor. This causes an increase in the frequency of chloride channel opening, leading to hyperpolarization of the cell membrane and reduced excitability of nerve cells [38]. Depending on the age of the child, the following doses are administered: infants from 7 months of age: up to 0.5 mg/kg body weight; children weighing 10–15 kg: one microinfusion (5 mg diazepam); children weighing more than 15 kg: two micro-infusions of 5 mg (10 mg diazepam) [38].

It is mainly administered as a rectal gel and as a nasal spray [39]. The efficacy of intranasal administration of diazepam has been tested. A total of 163 patients were included in the study with intranasal diazepam—the first group comprising 78 children aged 6 to 11 years, and the second group comprising 33 patients aged 12–17 years. Patients in the first group received one to eight doses per month of diazepam at 0.16–0.75 mg/kg, depending on their needs, of which 41 patients received the recommended dose of 0.3 mg/kg per month intranasally for 12 months, and the second group receivedone to six doses per month at 0.1–0.54 mg/kg for 12 months. Parents of the patients or the patients themselves, who were older, responded to a questionnaire, of which a total of 10 patients in group one and 10 patients in group 2 responded. The results of the questionnaire state that 84% of patients in both groups returned to normal function within 4 h of administration of the rescue medication, and 20% after 30 min. In addition, 88% of the patients confirm that they felt very good and comfortable with the diazepam spray while doing activities outside the home. Furthermore, 11-year-olds and 16-year-olds from both groups reported that self-administration during a seizure outside the home was very easy. However, adverse effects related to administration such as fever (17.9%) and upper respiratory tract infection (17.9%) were reported most frequently, with nosebleeds (2.6%) reported less frequently [40]. Diazepam intranasally should be used from 6 years of age up to 11 years of age; the dose is 0.3 mg/kg, with 0.2 mg/kg above 12 years of age [41,42].

Midazolam is another first-line drug used to treat epileptic seizures in patients. The mechanism of action of midazolam is indirect and involves increasing the concentration of GABA and its affinity for benzodiazepine receptors. GABA and benzodiazepine receptors are linked to the chloride channel, which they jointly regulate. Midazolam increases the frequency of the opening of these chloride channels. Activation of both receptors leads to hyperpolarization of the cell membrane and inhibition of neuronal activity. The anticonvulsant effect of midazolam is due to the increased effect of GABA on motor circuits in the brain [43]. In daily use, it is used in the following doses: intravenously (from 6 months to 5 years), starting dose: 0.05–0.1 mg/kg, total dose: <6 mg. Intravenously (6–12 years), starting dose: 0.025–0.05 mg/kg; total dose: <10 mg. Rectally (over 6 months): 0.3–0.5 mg/kg, intramuscularly (1–15 years): 0.05–0.15 mg/kg [43].

In children, it is mainly administered intravenously or intramuscularly, but intranasal administration is becoming increasingly popular. A total of 42 patients, aged 5–92 years, were treated with intranasal midazolam at a dose of 2.5–15 mg under EEG guidance. In 57.1% of the patients, after intranasal midazolam administration, the convulsive state resolved in the EEG recording—the patients had not previously taken another anticonvulsant drug, while in 15 patients, an increase in diffuse slowing could be seen in the EEG. Intranasal administration of midazolam was not associated with serious side effects or adverse reactions—only five patients reported nasal irritation. Compared with intravenous administration, the bioavailability of the nasal spray is approximately 72.5–83% [44]. 

In light of the studies presented here, the administration of benzodiazepines in the form of an intranasal spray is much easier for pediatric patients, is non-invasive, has a well-defined safety profile, and is as effective as the other routes of administration [45]. Table 3 lists studies proving the effectiveness of different doses of diazepam and midazolam intranasal in reducing seizures and adverse effects.

The use of diazepam and midazolam intranasally is still not as popular as previous routes of administration, but the results of the studies presented in the review show that this may become an alternative, particularly for younger patients, who confirm that using these drugs intranasally is definitely easier for them.

### 3.4. (-)-trans-cannabidiol (CBD)

For the treatment of epileptic seizures in children, the use of (-)-trans-cannabidiol (CBD), the main ingredient in cannabis, in addition to commonly used drugs, is also reported. In preclinical studies, highly purified CBD has been shown to reduce recurrent seizures and, in the course of acute seizures, to regulate the ratio of synaptic excitation to inhibition [46].

In a study in mice, CBD administration at a dose of 100–200 mg was shown to reduce seizure duration as well as seizure severity and frequency [47,48]. In the next study, rats were treated at doses of 1, 10, 50, 100, and 200 mg/kg CBD—Epidolex and vehicle (vehicle group), with 12 animals in each group (*n* = 12). The effectiveness of the drug was assessed on the 7th day after birth—this is intended to reflect neonatal seizures. The animals were subjected to electroshock therapy to induce seizures. In the vehicle group, the average duration of seizures was 25 s. In the groups with CBD doses of 1, 10, and 50 mg/kg, no significant reduction in the duration of seizures was observed, while in the groups with CBD doses of 100 and 200 mg/kg, this time was reduced to 13 and 10 s, respectively [49].

The mechanism of action still remains incompletely identified, which raises some contradictions. Children with epilepsy have changes in the function of the endocannabinoid system, which modulates the balance between excitatory and inhibitory neurotransmitters [50]. Hemp oil has antioxidant, anti-inflammatory, and neuroprotective effects, which is an important etiological factor in the development of epilepsy in children. CBD is thought to interact with ion channels, signaling proteins, and transporters, specifically two G-protein-coupled receptors (GCPRs): the cannabinoid receptor (CB1R) and the de-orphanized GPR55 receptor, in both children and adults [51].

These are among the key receptors in the pathophysiology of epilepsy, as GPCRs regulate the ratio of synaptic excitations to their inhibition by linking endocannabinoids to downstream signaling. It has been suggested that CBD is a negative allosteric modulator of CB1R, affects it weakly, and in most preclinical models, this mechanism causes seizures [52]. It is also an antagonist of the GPR55 receptor, which will involve preventing it from binding to the lysophosphatidylinositol (LPI) lipid receptor—an endogenous agonist [51,52]. 

LPI, by binding to the GPR55 receptor, causes an influx of Ca^2+^ ions into the nerve cell bodies and presynaptic nerve endings that stimulate CA3–CA1 of the hippocampus, subsequently causing neuronal excitation. By inhibiting this mechanism, CBD also blocks increased glutamate release and excessive synaptic discharge [50]. 

Another mechanism suggesting a therapeutic effect of CBD in epilepsy is that it affects levels of anandamide (AEA), an endocannabinoid that controls neuronal excitability [53].

To confirm this thesis, peripheral blood and cerebrospinal fluid were collected from 12 patients untreated for epilepsy, aged 27–72 years. After examination of the fluid in the laboratory, it was found that AEA levels in epileptic patients were 5-fold lower than in healthy patients—the highest AEA concentration in the healthy patients was 25 pmol/mL, while in the sick patients the highest level reached only just over 5 pmol/mL [54].

CBD administration in studies was correlated with an increase in AEA in the cerebrospinal fluid, by inhibiting the breakdown of AEA by preventing the transport of AEA from the postsynaptic membrane of the neuron to the fatty acid amide hydrolase, FAAH, which is located in the endoplasmic reticulum of the neuron [55]. 

The most recently discovered mechanism, is the desensitizing effect of CBD on non-cannabinoid transient receptor potential vanilloid 1 (TRPV1) and TRPV2 ion channels, which prevents the release of calcium ions and a consequent decrease in neuronal excitability [51].

In a randomized control study in children with Dravet syndrome—a severe myoclonic epilepsy affecting infants—12.5% of children receiving CBD for 14 weeks of treatment experienced no epileptic seizures, while in a group of 59 children receiving placebo, no child experienced a reduction in seizures [56].

The effect on the frequency of tonic-clonic seizures in children was also studied for 14 weeks—there was a reduction in the median frequency of tonic-clonic seizures in children with Dravet syndrome and in six children with tuberous sclerosis [56].

In children with Lennox-Gastaut syndrome—an epileptic encephalopathy in early childhood—in 37% (23 out of 86 patients in the group) of children assigned to the study group (with CBD administration), the number of epileptic seizures was reduced by 50%, compared to the control group taking placebo, where the number was only reduced by 21% [57].

From questionnaires completed by parents of epileptic children aged 2 to 16, it was learned that of 19 patients using CBD at doses ranging from 0.5 mg/kg per day to 28.6 mg/kg/d, depending on the child’s weight, 84% reported fewer seizures, and 11% of them got rid of seizures completely [58].

Another questionnaire, completed this time by 117 parents of sick children aged 3 to 10 years, including more than 40% with incurable epileptic encephalopathies, reports that 85% of those treated with CBD at an average dose of 4.3 mg/kg/d (if parents reported weight) responded to treatment by reducing seizure frequency, and 14% reduced this amount to zero [59].

The EMA approved using a highly purified CBD preparation—Epidiolex in conjunction with other drugs such as clobazam, valproid acid, lamotigine, and everolimus, and it is indicated for the treatment of severe epileptic syndromes, Dravet syndrome, Lennox-Gastaut syndrome, and tuberous sclerosis complex [60]. Generalized tonic-clonic seizures (GTC) in rats were induced by increasing the temperature by 0.5 °C every 2 min. The durations of febrile convulsions in the group with clonazepam at a dose of 0.0625 mg/kg and a combination of clonazepam with CBD (COM) at a dose of 100 mg/kg were examined. In the clonazepam-treated group of rats, the average seizure duration was 25.4 s (±5.2), in the CBD group—20.2 (±2) s, and in the COM group—13.8 (±3.5) s [61].

Additionally, COM increases the threshold temperature for thermal seizure induction. In the clonazepam group, the induction threshold was 39.4 (±0.27) s, in the CBD group—38.6 (±0.40) s, and in the COM group—39.0 (±0.28) s [61]. Table 4 lists summary of the effects of CBD in pediatric patients with Lennox-Gastaut syndrome and Dravet.

It is also said that CBD can be effective in treating psychiatric disorders in children and children on the autism spectrum. The use of medicinal cannabis in the treatment of developmental, behavioral, and mental health disorders in children is discussed in a recent Efron and Talyor review [51].

### 3.5. Brivaracetam

Brivaracetam (BRV) is classified as a third-generation ASD [64]. It was approved by the FDA and EMA in 2016 [65]. In terms of structure, it belongs to the 4-n-propyl analogue of levetiracetam (LEV) [50]. The substance’s mechanism lies in its high and selective affinity for synaptic vesicle glycoprotein 2A (SV2A), which is as much as 15–30 times higher compared to LEV. SV2A is among the transmembrane glycoproteins found at glutamatergic and GABAergic synaptic terminals, and thus has implications for neuromodulation and neurotransmission [65]. The protein regulates the release and reuptake of neurotransmitters, which allows a decrease in neuronal excitability in the brain, which is desirable in epilepsy therapy [66]. In addition, it is found to block voltage-dependent sodium channels in neurons [67]. 

BRV is one of the substances that rapidly penetrates the blood–brain barrier, and its bioavailability is almost 100% [66,68]. The drug is metabolized in the liver with the involvement of CYP450, particularly CYP2C9 and CYP2C19 [69,70]. Patients using BRV have been noted to experience headaches, dizziness, drowsiness, back pain, fatigue, and irritability [71].

BRV is a drug approved in the European Union for adjunctive therapy in patients aged 2 years and older, while in the United States it is approved for monotherapy as well as adjunctive treatment in children aged 1 month and older [69,72,73]. The drug is used in patients with focal seizures, generalized seizures, drug-resistant epilepsy, and epileptic syndromes [72,74,75,76].

BRV is used orally, and its dosage depends on the patient’s weight. Children weighing 11–20 kg get an initial dose of 1–2.5 mg/kg/d, while the maintenance dose is 5 mg/kg/d. Adequately, patients weighing 20–50 kg receive 0.5–1 mg/kg twice daily and 1–4 mg/kg/d. In contrast, patients weighing more than 50 kg receive 2 mg/kg, but the daily dose is a maximum of 100 mg taken twice [77].

The clinical experiment included 23 patients (14 girls and 9 boys) with drug-resistant epilepsy who were treated with BRV. The mean age of the patients was 12.5 years, while the drug dose received was 3.9 mg/kg/d. In eight patients, a reduction in seizure frequency of more than 50% was achieved. In addition, lethargy was noted in two children, and three had behavioral disturbances [78].

A retrospective and descriptive study involving 66 patients aged 1–16 years was performed. In this group, 27 children had epileptic encephalopathy, and 93.4% of patients suffered from drug-resistant epilepsy. In 30.3% of the patients, seizure frequency decreased by more than 50%, whereas 9% of the patients had no seizures at all. Drowsiness, irritability, and nervousness were observed in 24.2%. It was found that there was a better response to treatment in patients who were directly changed from LEV to BRV than in the portion whose therapy was gradually changed. The rates of better response to treatment were 66.7% and 24.1%, respectively [73].

The study, which ran from 2011 to 2022 to evaluate the long-term efficacy and safety of BRV as an adjunctive therapy, involved 257 patients under the age of 17. The study found that while taking the drug for such a long time, as many as 93.4% of patients developed adverse reactions. Among the most common were nasopharyngitis and pharyngitis (29.2% and 22.3%), fever (25.3%), vomiting (21.4%), headache (15.2%), decreased appetite (11.7%), or drowsiness (10.5%). In contrast, serious effects such as status epilepticus, pneumonia, and generalized tonic-clonic seizures were observed adequately in 4.3%, 3.1%, and 1.9% of patients. By analyzing the 28-day frequency of overall and focal seizures, a decrease in their occurrence was found in 87.7% and 96.9% of patients before 2 years of age, respectively, while an overall reduction in seizures was noted in 68.2% of children at this age. However, in patients after the age of 2 years, 60.3%, 63%, and 50.9% were similarly affected. The study proved that long-term use of BRV in children, although often associated with adverse symptoms, significantly reduced the incidence of focal or generalized seizures [79].

The study included 31 patients between the ages of 6 and 20 who had been switched to BRV due to a lack of response to previous treatments or adverse reactions to therapy. 20 of them had focal epilepsy, 11 had Lennox—Gastaut syndrome, 3 had myoclonic—atonic syndrome, and 3 had myoclonic absence. The results showed that 45% of all patients responded positively to treatment. In contrast, the number of patients who experienced side effects was low. Drowsiness was experienced by 6.4% of them, psychosis by 3.2%, and nausea occurred in 3.2% of patients [80]. A clinical experiment was performed to determine the efficacy of BRV use in 46 patients under the age of 18. The results were very promising, as 65% of the patients responded to the therapy, and 30% of them did not experience any seizures. Adverse effects such as drowsiness or irritability were observed in 43.5% of the subjects [81].

Based on a number of studies conducted on a group of pediatric patients, it can be concluded that BRV is a drug that effectively reduces the incidence of epileptic seizures. Although a sizable group of patients experienced side effects, they were relatively mild. BRV is a new drug that doctors will increasingly use in children affected by various types of epilepsy [73,75,78,79,80,81]. Table 5 summarizes the studies documenting the effectiveness of brivaracetam. 

### 3.6. Ganaxolone

Ganaxolone (GNX) is a new drug classified as a neuroactive GABAA receptor (GABAAR) modulator [82]. It is among substances with multiple potential actions: antiepileptic, antidepressant, anti-anxiety, sedative, stress-reducing, sleep-promoting, neuroprotective, anti-aggressive, pro-sexual, and pro-social [83]. GNX was approved for patients over 2 years of age by the FDA in March 2022 and by the EMA in May 2023.

GNX, or 3α-hydroxy-3β-methyl-5α-pregnan-20-one, is a 3β-methylated analog of the neurosteroid allopregnanolone, which is classified as a metabolite of progesterone [84]. Through methylation, there is no rapid metabolism of the drug, and the terminal half-life is 34 h [85]. It reaches its maximum plasma concentration 2 to 3 h after oral administration. The drug is metabolized in the liver mainly by CYP3A4/5, but also by CYP2B6, CYP2C19, and CYP2D6 [86].

The main inhibitory neurotransmitter in the central nervous system is γ-aminobutyric acid (GABA). Through activation of GABAAR at presynaptic and postsynaptic connections by GNX, there is an increase in suppression of neuronal excitability [85]. Neurosteroid binding to GABAAR causes an increase in chloride channel permeability. In addition, it is found that a low concentration (20 nM) of GNX activates membrane progesterone receptors (mPR), mainly δ, so that it has an anti-apoptotic effect on nerve cells. By activating inhibitory G protein (Gi)-dependent signaling through mPRα and/or mPRβ, there are specific messages about the neuroprotective effects of GNX [87].

For pediatric patients, GNX is administered orally, gradually increasing the dose three times a day with meals. In patients weighing less than 28 kg, the starting dose is 18 mg/kg/d, while the maximum dose is 63 mg/kg/d. In patients over 28 kg, the doses are 450 mg/d and 1800 mg/d [88].

The indication for GNX is cyclin-dependent kinase-like 5 (CDKL5) deficiency syndrome (CDD), consisting of severe early-onset epilepsy with associated multiple cognitive, motor, pattern, and autonomic disorders [82]. In addition, the drug’s efficacy has been studied in focal epilepsy, refractory status epilepticus, menstrual epilepsy, disorders associated with fragile X syndrome, and infantile spasms [89]. 

The study included 101 patients with CDD: 50 took GNX, 51 took placebo. In 24% of patients taking GNX, the incidence of major seizures was reduced by more than 50%, whereas in the placebo group it was reduced by 10%. Adverse symptoms occurred in 86% of the study group and 88% of the control group. The most common were lethargy, upper respiratory tract infections, or fever [90].

Based on available information on the therapy of 50 CDD patients taking GNX, the median 28-day decrease in the incidence of severe seizures was 48.2%. After 22–24 months of therapy, reductions in the incidence of severe seizures of more than 25%, 50%, 75%, and 100% were found in 66%, 46%, 24%, and 6% of patients, respectively. Based on an analysis of the results of 88 patients taking GNX, adverse symptoms were reported by 46.6% of them. A total of 17% experienced lethargy, 5.7% decreased appetite, 4.5% weight loss, 3.4% behavioral disturbances, and 3.4% gait disturbances [91].

The study included 21 girls between the ages of 5 and 10 with protocadherin-19 (PCDH19)-clustering epilepsy. The disease is caused by a mutation of the *PCDH19* gene located on the X chromosome. It is characterized by the occurrence of treatment-resistant epileptic seizures, intellectual disability, autism, and behavioral disorders. Based on the analysis, 50% of those taking GNX reported a decrease in seizure frequency of more than 50% within 28 days, whereas the percentage of patients in the placebo group was 36.4%. In addition, a significant improvement in health was reported by 55.6% of patients in the study group, compared to 9.1% in the control group. Adverse symptoms occurred in 70% of patients taking the drug, compared to 100% of those taking the placebo. The most commonly reported were drowsiness, mental confusion, excitement, and gastrointestinal distress [92].

Based on the available data and studies, it can be concluded that GNX is a drug with great therapeutic potential and application in pediatric neurology. The accessible information predicts the safety and efficacy of using the drug on pediatric patients for specific types of epileptic seizures [90,91,92,93,94]. Table 6 shows the effect of GNX on the frequency of epileptic seizures.

## 4. Discussion

Epilepsy is encountered in 4–10% of the pediatric population [6,7]. A high risk of epilepsy occurrence is characteristic of the infantile period due to genetic, metabolic, and perinatal causes [5]. A mutation in the *SCN1A* gene is characteristic of epilepsy development [9]. Significant in the treatment of epilepsy is preventing seizure duration exceeding 30 min, as there is a risk of irreversible complications. First-line medications for antiepileptic treatment are benzodiazepines, while second-line options include phenobarbital, phenytoin, levetiracetam, and sodium valproate [13].

It’s crucial to diagnose epilepsy in children as quickly as possible and determine the cause of its occurrence. It’s also important to promptly initiate appropriate epilepsy treatment, depending on its type, to prevent irreversible changes due to seizures.

CNB, acting as a modulator of GABAA receptors and blocking sodium channels, is approved only for the treatment of focal seizures in adults. However, it is not approved for use in children due to limited research [20,21,22,23]. Research conducted on a group of patients ranging from 10 to 18 years of age showed a complete reduction of seizures in 31.3% and 19% of patients, a decrease in the number of seizures by more than half in 37.5% and 52%, a reduction of seizures by at least 50% in 62% and 61.5%, and a reduction of seizures below 50% in 37.5% [20,23].

FFA, used in the treatment of DS and LGS from the age of 2, has also been recognized as a safe adjunctive therapy for other ASDs from the age of 2 and older children [28]. Studies conducted on patients with DS in age groups ranging from 2 to 18 years old showed that the use of FFA resulted in a 42.3% reduction in seizures, with the percentage of seizure reduction increasing to 74.9% as the dose increased. In another group of patients aged 6–19 years old, the reduction was over 50%. Additionally, in patients with the *SCN1A* mutation, a reduction in seizures of 50% was also demonstrated [31,32,33].

The studies conducted on fenfluramine clearly indicate a positive impact of introducing it into antiepileptic therapy in children, with a notable reduction in seizures and increased seizure-free periods.

Midazolam acts on glycine receptors and increases the rate of opening chloride channels [43]. Seizure cessation in an EEG was observed in 57.1% of patients following nasal administration of midazolam [44]. Nasal administration of benzodiazepines not only facilitates drug delivery but is also less invasive compared to other routes of administration. Studies have shown that this route of administration takes the least amount of time from administration to seizure cessation, is more effective than intramuscular administration of the drug, and has demonstrated the highest rank probability in total time with the second-highest successful cessation rate in the pediatric population [95]. 

CBD acts on ion channels, signaling proteins, transporters, and two GPCRs, the cannabinoid receptor CB1R and the GPR55 receptor. Cannabidiol also functions as a negative allosteric modulator of CB1R and an antagonist of the GPR55 receptor [46,53]. It has also been shown to increase the level of AEA in cerebrospinal fluid [56]. CBD desensitizes non-cannabinoid TRPV1 and TRPV2 ion channels, which prevents the release of calcium ions, thereby reducing neuronal excitability [66]. The research results showed that the use of CBD in patients with DS resulted in a lack of seizures in 12% of children over a period of 14 weeks, while patients with tuberous sclerosis also noticed a reduction in seizures. Patients with LGS experienced a 50% decrease in seizures [57,96,97,98,99].

From the studies conducted, it is evident that CBD holds great promise for pediatric patients in reducing or completely eliminating epileptic seizures.

It’s worth noting that the EMA has approved the combination of CBD with other medications. The medications in question are clobazam, valproate, stiripentol, lamotrigine, and everolimus. The use of these medications requires regular follow-up visits. The dosage of ASDs and CBD is determined individually for each patient during follow-up visits, with simultaneous monitoring of adverse effects and CBD plasma levels. This is due to the complicated pharmacokinetics of CBD, which may interact with the aforementioned medications. CBD is administered orally, with an initial dose of 5 mg/kg/d, increasing to 10 mg/kg/d after one week [60].

BRV acts through selective affinity to synaptic vesicle glycoprotein 2A (SV2A) and blocks voltage-dependent sodium channels in neurons [65,67]. It has been approved for use in monotherapy or adjunctive therapy in children aged 1 month and older in the USA and as adjunctive therapy in children aged 2 years and older in the EU [70,71,74]. The research demonstrated a reduction in seizure frequency by over 50% and even a complete cessation of seizures. In the group of patients up to 17 years of age, a decrease in seizures of 87.7% was observed, a decrease of 96.9% for focal seizures in children under 2 years of age, and a decrease of 68.2% in children aged 2 years. The medication exhibited efficacy in LGS, focal epilepsy, myoclonic-atonic syndrome, and myoclonic absence seizures [74,75,81].

The above studies demonstrate that, despite its high efficacy, the medication has adverse effects that do not occur in every patient. Therefore, when implementing BRV treatment, physicians should carefully monitor patients to determine whether the benefits of the treatment outweigh the adverse effects if they occur during treatment.

GNX is approved for use in patients over 2 years of age by the FDA and EMA. The medication activates GABAAR, causing increased neuronal excitability suppression, and exhibits neuroprotective effects [84,85,86]. GNX is administered for indications such as CDKL5 CDD [69]. The medication demonstrated a significant reduction in seizures in patients with CDD, cluster epilepsy, PCDH19, and treatment-resistant infantile spasms [73,80,81,82].

Analyzing all the above studies, it can be concluded that the drugs discussed exhibit significant efficacy. These medications demonstrated reductions or decreases in epileptic seizures and some extended seizure-free periods in children. Can we then determine which of them is the most effective and safest for children? Unfortunately, this is difficult to determine. Some of the discussed drugs have been approved by the FDA and EMA for use in children, such as GNX, diazepam and midazolam intranasally, CBD, BRV, and FFA, while others, like CNB, have not. Can it be assumed then that drugs approved by the FDA and EMA for use in children are the safest with high therapeutic efficacy? At present, this conclusion can be drawn. Alternative therapies for treating epilepsy include steroid agents, immunosuppressive agents, electrical and magnetic stimulation therapies, the ketogenic diet, pyridoxine infusion, cerebrospinal fluid drainage, and magnesium infusion [100].

Clinical trials are currently being conducted on the safety of using CNB, the long-term safety, tolerance, and efficacy of BRV, the safety, tolerance, and pharmacokinetics of FFA in infants aged 1 year to under 2 years with Dravet syndrome, and phase III studies on adjunctive treatment with GNX [101,102,103,104].

The studies discussed in this article have several limitations. Firstly, the studies we relied on were conducted in small study groups, and not every study was limited to individuals under 18 years of age. Another limitation of our study was the lack of information in the studies we relied on regarding whether the drugs were administered as monotherapy or in combination with other medications.

Therefore, in our opinion, it is necessary to conduct studies on a larger, strictly defined age group of children and demonstrate whether it will be possible to administer the preparations in combination with other drugs or in monotherapy. It will also be important to verify whether, as a result of using the discussed drugs in combination with other preparations, it will be possible to reduce the dosage of the drugs and to investigate the safety of their long-term therapy. Prospects for the development of ASDs should focus on obtaining drugs with easy administration routes for children or parents and reducing adverse effects. This will enable the modification of epilepsy treatment in children to achieve longer seizure-free periods or a complete reduction to zero.

## Figures and Tables

**Table 1:** Studies describing the effectiveness of cenobamate in the reduction of seizures.

Author of the Study	Year of Publication	Number of Patients	Age of Patients	Dose	≤50% Seizure Reduction	>50% Seizure Reduction	Complete Seizure Reduction	Adverse Effects	Type of Epilepsy
Makridis KL et al. [23]	2022	16	12–18	6.25–25 mg/d	6 (37.5%)	6 (37.5%)	5 (31.3%)	somnolence, fatigue	Drug-resistant epilepsy
Elliott et al. [27]	2022	13	12–17	50–300 mg/d	no data	8 (61.5%)	no data	somnolence	Focal-onset epilepsy
Varughese RT et al. [20]	2022	21	10–18	200 mg/d	13 (61.9%)	11 (52.3%)	4 (19%)	somnolence, fatigue	Focal-onset epilepsy
				209.8 mg/d					

**Table 2:** Studies describing the effectiveness of different doses of fenfluramine in reducing seizures compared to placebo.

Author of the Study	Year of Publication	Number of Patients	Age of Patients	Dose	% of Seizure Reduction Compared to Placebo	Adverse Effects	Type of Epilepsy
Nabbout et al. [31]	2020	87	6–19	0.4 mg/kg/d	54%	decreased appetite, fever, fatigue, diarrhea	Dravet syndrome
Lagae et al. [32]	2019	119	2–18	0.2 mg/kg/d	42.30%	decreased appetite, fever, fatigue, diarrhea	Dravet syndrome
				0.7 mg/kg/d	74.90%		
Specchio N et al. [33]	2020	52	4–14	0.2–0.7 mg/kg/d	77.40%	decreased appetite	Dravet syndrome
Knupp et al. [36]	2023	168	2–18	0.2–0.7 mg/kg/d	25.60%	decreased appetite, fatigue	Dravet syndrome

**Table 3:** Studies describing the effectiveness of different doses of diazepam and midazolam intranasal in reducing seizures and adverse effects.

Author of the Study	Year of Publication	Number of Patients	Age of Patients	Dose	Positive Response to the Treatment	Adverse Effects
Tarquinio D et al. [40]	2022	163	12–17	0.3 mg/kg	143 (88%)	fever (17.9%), upper respiratory tract infection (17.9%), nosebleeds (2.6%)
Kay L et al. [44]	2019	42	5–92	2.5–15 mg/kg	24 (57.1%)	nasal irritation (11.9%)

**Table 4:** Summary of the effects of CBD in pediatric patients with Lennox-Gastaut syndrome and Dravet syndrome.

Author of the Study	Year of Publication	Number of Patients	Age of Patients	Dose	Positive Response to the Treatment	Adverse Effects	Type of Epilepsy
Elliott J. et al. [51]	2019	19	2–16	0.5–28.6 mg/kg	16 (84%)	vomiting, diarrhoea	Lennox-Gastaut syndrome
Thiele E. et al. [57]	2018	86	2–17	10–20 mg/kg	23 (37%)	diarrhea, lethargy, fever, decreased appetite, and vomiting	Lennox-Gastaut syndrome
Devinsky O. et al. [62]	2018	73 76	2–55	10 mg/kg 20 mg/kg	27 (37.2%) 32 (41.9%)	vomiting, diarrhea, fatigue, somnolence, pyrexia	Dravet syndrome
Miller et al. [63]	2020	66 67	2–18	10 mg/kg 20 mg/kg	30 (47.8%) 30 (45.7%)	decreased appetite, diarrhea, somnolence, pyrexia, fatigue	Dravet syndrome

**Table 5:** Studies describing responses to brivaracetam treatment and its adverse effects.

Author of the Study	Year of Publication	Number of Patients	Age of Patients	Dose	Type of Epilepsy	Positive Response to the Treatment	Adverse Effects
McGuire S et al. [78]	2020	20	4–20	3.9 mg/kg/d	refractory epilepsy	9 (45%)	psychiatric adverse effects (17%)
Lagae L et al. [79]	2023	257	1 month–17 years	5 mg/kg/d	focal epilepsy, generalized epilepsy, generalized epileptic syndrome or other symptomatic generalized epilepsy	225 (87.7%)	inflammation of the nasopharynx (29.2%); inflammation of the throat (22.3%); fever (25.3%); vomiting (21.4%); headache (15.2%); decreased appetite (11.7%); abdominal pain (10.5%); status epilepticus (4.3%); pneumonia (3.1%); generalized tonic-clonic seizures (1.9%)
Nissenkorn A et al. [80]	2019	31	6–20	100–300 mg	focal epilepsy, Lennox- Gastaut syndrome, myoclonic- atonic syndrome, myoclonic absence	14 (45%)	drowsiness (6.4%); nausea (3.2%); psychosis (3.2%)
Visa-Reñé N et al. [81]	2020	46	0–18	initial average 2.8 mg/kg/d	focal epilepsy, generalized epilepsy	30 (65%)	drowsiness and irritability (43.5%)

**Table 6:** Studies describing the effectiveness of ganaxolone in reducing seizure frequency (CDD—CDKL5 deficiency disorder, PCDH19—Protocadherin-19, CDKL5—cyclin-dependent kinase-like 5).

Author of the Study	Year of Publication	Number of Patients	Age of Patients	Dose	Type of Epilepsy	Seizure Frequency Reduction	Adverse Effects
Knight et al. [90]	2022	50	2–21	63 mg/kg ≤28 kg 1800 mg/d > 28kg	CDD	50%	somnolence (36%), pyrexia (18%), seizure (14%), vomiting (10%), upper respiratory tract infection (10%), constipation (6%), sedation (6%), salivary hypersecretion (6%), ear infection (4%), rash (4%)
Olson HE et al. [91]	2024	50	2–19	63 mg/kg ≤28 kg 1800 mg/d > 28kg	CDD	48.20%	somnolence (17%), seizure (11.4%), decreased appetite (5.7%), pneumonia (5.7%) weight decrease (4.5%), attention-seeking behavior (3.4%), acute respiratory failure (3.4%), pneumonia aspiration (3.4%) gait disturbance (3.4%)
Sullivan J et al. [92]	2023	21	1–17	63 mg/kg ≤28 kg 1800 mg/d > 28kg	PCDH19—clustering epilepsy	55.60%	psychiatric disorders (50%), somnolence (40%), agitation (20%), gastrointestinal disorders (20%), diarrhea (10%), agression (10%), ataxia (10%)

## References

[1] Fisher R.S., Boas W.V.E., Blume W., Elger C., Genton P., Lee P., Engel J. (2005). Epileptic Seizures and Epilepsy: Definitions Proposed by the International League Against Epilepsy (ILAE) and the International Bureau for Epilepsy (IBE). Epilepsia.

[2] https://epilepsydiagnosis.org.

[3] Auvin S. (2022). Paediatric epilepsy and cognition. Dev. Med. Child Neurol..

[4] Bajaj J., Soni P., Khandelwal N., Hedaoo K., Kumar A., Sinha M., Ratre S., Parihar V., Swamy M.N., Yadav Y.R. (2022). Epilepsy-Related Injuries in Children: An Institution-Based Study. Neurol. India.

[5] Ioannou P., Foster D.L., Sander J.W., Dupont S., Gil-Nagel A., Drogon O’Flaherty E., Alvarez-Baron E., Medjedovic J. (2022). The burden of epilepsy and unmet need in people with focal seizures. Brain Behav..

[6] Sartori S., Nosadini M., Tessarin G., Boniver C., Frigo A.C., Toldo I., Bressan S., Da Dalt L. (2019). First-ever convulsive seizures in children presenting to the emergency department: Risk factors for seizure recurrence and diagnosis of epilepsy. Dev. Med. Child Neurol..

[7] Symonds J.D., Elliott K.S., Shetty J., Armstrong M., Brunklaus A., Cutcutache I., Diver L.A., Dorris L., Gardiner S., Jollands A. (2021). Early childhood epilepsies: Epidemiology, classification, aetiology, and socio-economic determinants. Brain J. Neurol..

[8] Ruggiero S.M., Xian J., Helbig I. (2023). The current landscape of epilepsy genetics: Where are we, and where are we going?. Curr. Opin. Neurol..

[9] Brunklaus A., Brünger T., Feng T., Fons C., Lehikoinen A., Panagiotakaki E., Vintan M.A., Symonds J., Andrew J., Arzimanoglou A. (2022). The gain of function SCN1A disorder spectrum: Novel epilepsy phenotypes and therapeutic implications. Brain J. Neurol..

[10] Rochtus A., Olson H.E., Smith L., Keith L.G., El Achkar C., Taylor A., Mahida S., Park M., Kelly M., Shain C. (2020). Genetic diagnoses in epilepsy: The impact of dynamic exome analysis in a pediatric cohort. Epilepsia.

[11] Hong W., Haviland I., Pestana-Knight E., Weisenberg J.L., Demarest S., Marsh E.D., Olson H.E. (2022). CDKL5 Deficiency Disorder-Related Epilepsy: A Review of Current and Emerging Treatment. CNS Drugs.

[12] Pisani F., Spagnoli C., Falsaperla R., Nagarajan L., Ramantani G. (2021). Seizures in the neonate: A review of etiologies and outcomes. Seizure.

[13] Messahel S., Bracken L., Appleton R. (2022). Optimal Management of Status Epilepticus in Children in the Emergency Setting: A Review of Recent Advances. Open Access Emerg. Med..

[14] Sculier C., Gaspard N. (2019). New onset refractory status epilepticus (NORSE). Seizure.

[15] Bjurulf B., Reilly C., Sigurdsson G.V., Thunström S., Kolbjer S., Hallböök T. (2022). Dravet syndrome in children-A population-based study. Epilepsy Res..

[16] Asadi-Pooya A.A. (2018). Lennox-Gastaut syndrome: A comprehensive review. Neurol. Sci..

[17] Nelson J.A., Knupp K.G. (2023). Lennox-Gastaut Syndrome: Current Treatments, Novel Therapeutics, and Future Directions. Neurotherapeutics.

[18] Barnett J.R., Fleming B.M., Geenen K.R., Sourbron J., Freedman J.H., Bruno P.L., Thiele E.A. (2020). Characterizing Sunflower syndrome: A clinical series. Epileptic Disord..

[19] Engel J. (2018). The current place of epilepsy surgery. Curr. Opin. Neurol..

[20] Varughese R.T., Shah Y.D., Karkare S., Kothare S.V. (2022). Adjunctive use of cenobamate for pediatric refractory focal-onset epilepsy: A single-center retrospective study. Epilepsy Behav..

[21] Sharma R., Nakamura M., Neupane C., Jeon B.H., Shin H., Melnick S.M., Glenn K.J., Jang I.S., Park J.B. (2020). Positive allosteric modulation of GABAA receptors by a novel antiepileptic drug cenobamate. Eur. J. Pharmacol..

[22] Roberti R., De Caro C., Iannone L.F., Zaccara G., Lattanzi S., Russo E. (2021). Pharmacology of Cenobamate: Mechanism of Action, Pharmacokinetics, Drug–Drug Interactions and Tolerability. CNS Drugs.

[23] Makridis K.L., Bast T., Prager C., Kovacevic-Preradovic T., Bittigau P., Mayer T., Breuer E., Kaindl A.M. (2022). Real-World Experience Treating Pediatric Epilepsy Patients with Cenobamate. Front. Neurol..

[24] Rissardo J.P., Fornari Caprara A.L. (2023). Cenobamate (YKP3089) and Drug-Resistant Epilepsy: A Review of the Literature. Medicina.

[25] Nakamura M., Cho J.H., Shin H., Jang I.S. (2019). Effects of cenobamate (YKP3089), a newly developed anti-epileptic drug, on voltage-gated sodium channels in rat hippocampal CA3 neurons. Eur. J. Pharmacol..

[26] Sankar R. (2023). Treatment of status epilepticus: Physiology, pharmacology, and future directions. Epilepsia Open.

[27] Elliott T., Ridley-Pryor T., Gienapp A.J., Wheless J.W. (2022). Initial Real-World Experience With Cenobamate in Adolescents and Adults: A Single Center Experience. Pediatr. Neurol..

[28] Tabaee Damavandi P., Fabin N., Giossi R., Matricardi S., Del Giovane C., Striano P., Meletti S., Brigo F., Trinka E., Lattanzi S. (2023). Efficacy and Safety of Fenfluramine in Epilepsy: A Systematic Review and Meta-analysis. Neurol. Ther..

[29] Samanta D. (2022). Fenfluramine: A Review of Pharmacology, Clinical Efficacy, and Safety in Epilepsy. Children.

[30] Sourbron J., Lagae L. (2022). Serotonin receptors in epilepsy: Novel treatment targets?. Epilepsia Open.

[31] Nabbout R., Mistry A., Zuberi S., Villeneuve N., Gil-Nagel A., Sanchez-Carpintero R., Stephani U., Laux L., Wirrell E., Knupp K. (2020). Fenfluramine for Treatment-Resistant Seizures in Patients With Dravet Syndrome Receiving Stiripentol-Inclusive Regimens: A Randomized Clinical Trial. JAMA Neurol..

[32] Lagae L., Sullivan J., Knupp K., Laux L., Polster T., Nikanorova M., Devinsky O., Cross J.H., Guerrini R., Talwar D. (2019). Fenfluramine hydrochloride for the treatment of seizures in Dravet syndrome: A randomised, double-blind, placebo-controlled trial. Lancet.

[33] Specchio N., Pietrafusa N., Doccini V., Trivisano M., Darra F., Ragona F., Cossu A., Spolverato S., Battaglia D., Quintiliani M. (2020). Efficacy and safety of Fenfluramine hydrochloride for the treatment of seizures in Dravet syndrome: A real-world study. Epilepsia.

[34] Gil-Nagel A., Sullivan J., Ceulemans B., Wirrell E., Devinsky O., Nabbout R., Knupp K.G., Scott Perry M., Polster T., Davis R. (2021). Treatment with fenfluramine in patients with Dravet syndrome has no long-term effects on weight and growth. Epilepsy Behav..

[35] Lattanzi S., Trinka E., Russo E., Del Giovane C., Matricardi S., Meletti S., Striano P., Damavandi P.T., Silvestrini M., Brigo F. (2023). Pharmacotherapy for Dravet Syndrome: A Systematic Review and Network Meta-Analysis of Randomized Controlled Trials. Drugs.

[36] Knupp K.G., Scheffer I.E., Ceulemans B., Sullivan J., Nickels K.C., Lagae L., Guerrini R., Zuberi S.M., Nabbout R., Riney K. (2023). Fenfluramine provides clinically meaningful reduction in frequency of drop seizures in patients with Lennox–Gastaut syndrome: Interim analysis of an open-label extension study. Epilepsia.

[37] Patel S., Geenen K.R., Dowless D., Bruno P.L., Thiele E.A. (2023). Follow-up to low-dose fenfluramine for Sunflower syndrome: A non-randomized controlled trial. Dev. Med. Child Neurol..

[38] Dhaliwal J.S., Rosani A., Saadabadi A. (2024). Diazepam. StatPearls [Internet].

[39] Becker D.A., Wheless J.W., Sirven J., Tatum W.O., Rabinowicz A.L., Carrazana E. (2023). Treatment of Seizure Clusters in Epilepsy: A Narrative Review on Rescue Therapies. Neurol. Ther..

[40] Tarquinio D., Dlugos D., Wheless J.W., Desai J., Carrazana E., Rabinowicz A.L. (2022). Safety of Diazepam Nasal Spray in Children and Adolescents with Epilepsy: Results from a Long-Term Phase 3 Safety Study. Pediatr. Neurol..

[41] Wheless J.W., Hogan R.E., Davis C.S., Carrazana E., Rabinowicz A.L. (2024). Safety and effectiveness of diazepam nasal spray in male and female patients: Post hoc analysis of data from a phase 3 safety study. Epilepsia Open.

[42] Cornett E.M., Amarasinghe S.N., Angelette A., Abubakar T., Kaye A.M., Kaye A.D., Neuchat E.E., Urits I., Viswanath O. (2021). VALTOCO^®^ (Diazepam Nasal Spray) for the Acute Treatment of Intermittent Stereotypic Episodes of Frequent Seizure Activity. Neurol. Int..

[43] Lingamchetty T.N., Hosseini S.A., Saadabadi A. (2024). Midazolam. StatPearls.

[44] Kay L., Merkel N., Von Blomberg A., Willems L.M., Bauer S., Reif P.S., Schubert-Bast S., Rosenow F., Strzelczyk A. (2019). Intranasal midazolam as first-line inhospital treatment for status epilepticus: A pharmaco-EEG cohort study. Ann. Clin. Transl. Neurol..

[45] Peters J., Becker D., Misra S., Carrazana E., Rabinowicz A. (2024). Taking a Newer, Faster, Intranasal Route: A Narrative Review of Transitioning to a Less-Invasive Rescue Treatment for Seizure Clusters. Patient Prefer. Adherence.

[46] Rosenberg E.C., Chamberland S., Bazelot M., Nebet E.R., Wang X., McKenzie S., Jain S., Greenhill S., Wilson M., Marley N. (2023). Cannabidiol modulates excitatory-inhibitory ratio to counter hippocampal hyperactivity. Neuron.

[47] Kaplan J.S., Stella N., Catterall W.A., Westenbroek R.E. (2017). Cannabidiol attenuates seizures and social deficits in a mouse model of Dravet syndrome. Proc. Natl. Acad. Sci. USA.

[48] Shapiro L., Escayg A., Wong J.C. (2022). Cannabidiol Increases Seizure Resistance and Improves Behavior in an Scn8a Mouse Model. Front. Pharmacol..

[49] Witherspoon E., Quinlan S., Forcelli P.A. (2022). Preclinical efficacy of cannabidiol for the treatment of early-life seizures. Pharmacol. Rep..

[50] de Oliveira F.R., da Silva N.M., Hamoy M., Crespo-López M.E., Ferreira I.M., da Silva E.O., de Matos Macchi B., do Nascimento J.L.M. (2022). The GABAergic System and Endocannabinoids in Epilepsy and Seizures: What Can We Expect from Plant Oils?. Molecules.

[51] Efron D., Taylor K. (2023). Medicinal Cannabis for Paediatric Developmental, Behavioural and Mental Health Disorders. Int. J. Environ. Res. Public Health.

[52] Kwan Cheung K.A., Peiris H., Wallace G., Holland O.J., Mitchell M.D. (2019). The Interplay between the Endocannabinoid System, Epilepsy and Cannabinoids. Int. J. Mol. Sci..

[53] Colangeli R., Morena M., Pittman Q.J., Hill M.N., Teskey G.C. (2020). Anandamide Signaling Augmentation Rescues Amygdala Synaptic Function and Comorbid Emotional Alterations in a Model of Epilepsy. J. Neurosci. Off. J. Soc. Neurosci..

[54] Romigi A., Bari M., Placidi F., Marciani M.G., Malaponti M., Torelli F., Izzi F., Prosperetti C., Zannino S., Corte F. (2010). Cerebrospinal fluid levels of the endocannabinoid anandamide are reduced in patients with untreated newly diagnosed temporal lobe epilepsy. Epilepsia.

[55] Henson J., Vitetta L., Quezada M., Hall S. (2021). Enhancing Endocannabinoid Control of Stress with Cannabidiol. J. Clin. Med..

[56] Elliott J., DeJean D., Clifford T., Coyle D., Potter B.K., Skidmore B., Alexander C., Repetski A.E., Shukla V., McCoy B. (2019). Cannabis-based products for pediatric epilepsy: A systematic review. Epilepsia.

[57] Thiele E.A., Marsh E.D., French J.A., Mazurkiewicz-Beldzinska M., Benbadis S.R., Joshi C., Lyons P.D., Taylor A., Roberts C., Sommerville K. (2018). Cannabidiol in patients with seizures associated with Lennox-Gastaut syndrome (GWPCARE4): A randomised, double-blind, placebo-controlled phase 3 trial. Lancet.

[58] Porter B.E., Jacobson C. (2013). Report of a parent survey of cannabidiol-enriched cannabis use in pediatric treatment-resistant epilepsy. Epilepsy Behav..

[59] Hussain S.A., Zhou R., Jacobson C., Weng J., Cheng E., Lay J., Hung P., Lerner J.T., Sankar R. (2015). Perceived efficacy of cannabidiol-enriched cannabis extracts for treatment of pediatric epilepsy: A potential role for infantile spasms and Lennox-Gastaut syndrome. Epilepsy Behav..

[60] Ammendolia I., Mannucci C., Cardia L., Calapai G., Gangemi S., Esposito E., Calapai F. (2023). Pharmacovigilance on cannabidiol as an antiepileptic agent. Front. Pharmacol..

[61] Chuang S.H., Westenbroek R.E., Stella N., Catterall W.A. (2021). Combined Antiseizure Efficacy of Cannabidiol and Clonazepam in a Conditional Mouse Model of Dravet Syndrome. J. Exp. Neurol..

[62] Devinsky O., Cross J.H., Laux L., Marsh E., Miller I., Nabbout R., Scheffer I.E., Thiele E.A., Wright S., Cannabidiol in Dravet Syndrome Study Group (2017). Trial of Cannabidiol for Drug-Resistant Seizures in the Dravet Syndrome. N. Engl. J. Med..

[63] Miller I., Scheffer I.E., Gunning B., Sanchez-Carpintero R., Gil-Nagel A., Perry M.S., Saneto R.P., Checketts D., Dunayevich E., Knappertz V. (2020). Dose-Ranging Effect of Adjunctive Oral Cannabidiol vs Placebo on Convulsive Seizure Frequency in Dravet Syndrome: A Randomized Clinical Trial. JAMA Neurol..

[64] Makke Y., Abou-Khalil B. (2019). Brivaracetam efficacy and safety in focal epilepsy. Expert Rev. Neurother..

[65] Wu P.P., Cao B.R., Tian F.Y., Gao Z.B. (2023). Development of SV2A Ligands for Epilepsy Treatment: A Review of Levetiracetam, Brivaracetam, and Padsevonil. Neurosci. Bull..

[66] Latimer D., Le D., Falgoust E., Abd-Elsayed A., Cornett E.M., Singh R., Choi J., Varrassi G., Kaye A.M., Kaye A.D. (2023). Brivaracetam to Treat Partial Onset Seizures in Adults. Health Psychol. Res..

[67] Russo A., Pruccoli J., Cesaroni C.A., Ingraffia P., Abd-Elsayed A., Cornett E.M., Singh R., Choi J., Varrassi G., Kaye A.M. (2022). Brivaracetam add-on treatment in pediatric patients with severe drug-resistant epilepsy: Italian real-world evidence. Seizure Eur. J. Epilepsy.

[68] Lee K., Klein P., Dongre P., Choi E.J., Rhoney D.H. (2022). Intravenous Brivaracetam in the Management of Acute Seizures in the Hospital Setting: A Scoping Review. J. Intensive Care Med..

[69] Stockis A., Nicolas J., Sargentini-Maier M.L., Krauwinkel W. (2023). Pharmacokinetics, Safety, and Tolerability of Brivaracetam in Healthy Elderly Participants. Clin. Pharmacol. Drug Dev..

[70] Moseley B.D., Chanteux H., Nicolas J.M., Laloyaux C., Gidal B., Stockis A. (2020). A review of the drug-drug interactions of the antiepileptic drug brivaracetam. Epilepsy Res..

[71] Khilari M., Nair P., Jha B. (2021). Brivaracetam: How Well Does It Fare as an Anti-Epileptic? A Review. Neurol. India.

[72] https://www.ema.europa.eu/en/documents/overview/briviact-epar-medicine-overview_en.pdf.

[73] Ferragut Ferretjans F., Soto Insuga V., Bernardino Cuesta B., Cantarín Extremera V., Duat Rodriguez A., Legido M.J., González Alguacil E., Furones García M., Gutiérrez Solana L., Moreno Cantero T. (2021). Efficacy of Brivaracetam in children with epilepsy. Epilepsy Res..

[74] Lattanzi S., Canafoglia L., Canevini M.P., Casciato S., Cerulli Irelli E., Chiesa V., Dainese F., De Maria G., Didato G., Di Gennaro G. (2023). Adjunctive brivaracetam and sustained seizure frequency reduction in very active focal epilepsy. Epilepsia.

[75] Tulli E., Di Cara G., Iapadre G., Striano P., Verrotti A. (2021). An update on brivaracetam for the treatment of pediatric partial epilepsy. Expert Opin. Pharmacother..

[76] Verrotti A., Grasso E.A., Cacciatore M., Matricardi S., Striano P. (2021). Potential role of brivaracetam in pediatric epilepsy. Acta Neurol. Scand..

[77] Schoemaker R., Wade J.R., Stockis A. (2017). Brivaracetam population pharmacokinetics in children with epilepsy aged 1 month to 16 years. Eur. J. Clin. Pharmacol..

[78] McGuire S., Silva G., Lal D., Khurana D.S., Legido A., Hasbani D., Carvalho K.S., Melvin J., Valencia I. (2020). Safety and Efficacy of Brivaracetam in Pediatric Refractory Epilepsy: A Single-Center Clinical Experience. J. Child Neurol..

[79] Lagae L., Klotz K.A., Fogarasi A., Floricel F., Reichel C., Elshoff J.P., Fleyshman S., Kang H. (2023). Long-term safety and efficacy of adjunctive brivaracetam in pediatric patients with epilepsy: An open-label, follow-up trial. Epilepsia.

[80] Nissenkorn A., Tzadok M., Bar-Yosef O., Ben-Zeev B. (2019). Treatment with brivaracetam in children—The experience of a pediatric epilepsy center. Epilepsy Behav..

[81] Visa-Reñé N., Raspall-Chaure M., Paredes-Carmona F., Coromina J.S., Macaya-Ruiz A. (2020). Clinical experience with brivaracetam in a series of 46 children. Epilepsy Behav..

[82] Benke T.A., Demarest S., Angione K., Downs J., Leonard H., Saldaris J., Marsh E.D., Olson H., Haviland I., Adam M.P., Feldman J., Mirzaa G.M., Pagon R.A., Wallace S.E., Bean L.J.H., Gripp K.W., Amemiya A. (1993). CDKL5 Deficiency Disorder. GeneReviews^®^.

[83] Fasipe O.J., Agede O.A., Enikuomehin A.C. (2021). Announcing the novel class of GABA–A receptor selective positive allosteric modulator antidepressants. Future Sci. OA.

[84] Perucca E., Bialer M., White H.S. (2023). New GABA-Targeting Therapies for the Treatment of Seizures and Epilepsy: I. Role of GABA as a Modulator of Seizure Activity and Recently Approved Medications Acting on the GABA System. CNS Drugs.

[85] Yasmen N., Sluter M.N., Yu Y., Jiang J. (2023). Ganaxolone for management of seizures associated with CDKL5 deficiency disorder. Trends Pharmacol. Sci..

[86] De S.K. (2024). Ganaxolone: First FDA-approved Medicine for the Treatment ofSeizures Associated with Cyclin-dependent Kinase-like 5 DeficiencyDisorder. Curr. Med. Chem..

[87] Thomas P., Pang Y. (2020). Anti-apoptotic Actions of Allopregnanolone and Ganaxolone Mediated Through Membrane Progesterone Receptors (PAQRs) in Neuronal Cells. Front. Endocrinol..

[88] (2022). Ganaxolone. Am. J. Health Syst. Pharm..

[89] Samanta D. (2020). PCDH19-Related Epilepsy Syndrome: A Comprehensive Clinical Review. Pediatr. Neurol..

[90] Knight E.M.P., Amin S., Bahi-Buisson N., Benke T.A., Cross J.H., Demarest S.T., Olson H.E., Specchio N., Fleming T.R., Aimetti A.A. (2022). Safety and efficacy of ganaxolone in patients with CDKL5 deficiency disorder: Results from the double-blind phase of a randomised, placebo-controlled, phase 3 trial. Lancet Neurol..

[91] Olson H.E., Amin S., Bahi-Buisson N., Devinsky O., Marsh E.D., Pestana-Knight E., Rajaraman R.R., Aimetti A.A., Rybak E., Kong F. (2024). Long-term treatment with ganaxolone for seizures associated with cyclin-dependent kinase-like 5 deficiency disorder: Two-year open-label extension follow-up. Epilepsia.

[92] Sullivan J., Gunning B., Zafar M., Guerrini R., Gecz J., Kolc K.L., Zhao Y., Gasior M., Aimetti A.A., Samanta D. (2023). Phase 2, placebo-controlled clinical study of oral ganaxolone in PCDH19-clustering epilepsy. Epilepsy Res..

[93] Moncayo J.A., Vargas M.N., Castillo I., Granda P.V., Duque A.M., Argudo J.M., Matcheswalla S., Lopez Dominguez G.E., Monteros G., Andrade A.F. (2022). Adjuvant Treatment for Protocadherin 19 (PCDH19) Syndrome. Cureus.

[94] Perry M.S. (2020). New and Emerging Medications for Treatment of Pediatric Epilepsy. Pediatr. Neurol..

[95] Hasan S.U., Pervez A., Bhatty S., Shamim S., Naeem A., Naseeb M.W. (2022). Termination of seizures in the paediatric age group, best benzodiazepine and route of administration: A network meta-analysis. Eur. J. Neurosci..

[96] Thiele E.A., Bebin E.M., Filloux F., Kwan P., Loftus R., Sahebkar F., Sparagana S., Wheless J. (2022). Long-term cannabidiol treatment for seizures in patients with tuberous sclerosis complex: An open-label extension trial. Epilepsia.

[97] Chilcott E., Díaz J.A., Bertram C., Berti M., Karda R. (2022). Genetic therapeutic advancements for Dravet Syndrome. Epilepsy Behav..

[98] Strzelczyk A., Schubert-Bast S. (2021). Expanding the Treatment Landscape for Lennox-Gastaut Syndrome: Current and Future Strategies. CNS Drugs.

[99] Ben-Zeev B. (2020). Medical Cannabis for Intractable Epilepsy in Childhood: A Review. Rambam Maimonides Med. J..

[100] Kirmani B.F., Au K., Ayari L., John M., Shetty P., Delorenzo R.J. (2021). Super-Refractory Status Epilepticus: Prognosis and Recent Advances in Management. Aging Dis..

[101] Sunita Misra, Cenobamate Open-Label Extension Study for YKP3089C025. https://clinicaltrials.gov./study/NCT03961568?cond=Cenobamate&aggFilters=ages:child&rank=1.

[102] UCB Cares A Study to Test the Long-Term Safety, Tolerability and Efficacy of Brivaracetam in Study Participants 2 to 26 Years of Age with Childhood Absence Epilepsy or Juvenile Absence Epilepsy. https://clinicaltrials.gov./study/NCT05109234?cond=Brivaracetam&aggFilters=ages:child,status:enr&rank=1.

[103] UCB Cares A Study to Evaluate Safety, Tolerability, and Pharmacokinetics of Fenfluramine (Hydrochloride) in Infants 1 Year to Less Than 2 Years of Age with Dravet Syndrome (ORCHID). https://clinicaltrials.gov./study/NCT06118255?cond=Fenfluramine&aggFilters=ages:child&rank=7.

[104] Marinus Pharmaceuticals Adjunctive GNX Treatment Compared with Placebo in Children and Adults with TSC-Related Epilepsy. https://clinicaltrials.gov./study/NCT05323734?cond=Ganaxolone&aggFilters=ages:child&page=2&rank=13.

